# The Involvement of Ceramide, Sphingosine-1-Phosphate and Ganglioside GM1 in Regulating Some Nervous System Functions

**DOI:** 10.3390/ijms262211118

**Published:** 2025-11-17

**Authors:** Paola Giussani, Laura Mauri, Sandro Sonnino

**Affiliations:** Department of Medical Biotechnology and Translational Medicine, Università degli Studi di Milano, LITA Segrate, Via Fratelli Cervi, 93, 20054 Segrate, Italy

**Keywords:** sphingolipids, ceramide, sphingosine-1-phosphate, glycosphingolipids, gangliosides, nervous system

## Abstract

Sphingolipids are a large group of molecules, crucial components of all mammalian cells, that are particularly abundant in the central and peripheral nervous system and associated with important human brain functions. Sphingolipids are necessary for membrane organization and driving functions. Ceramide, sphingosine-1-phosphate and GM1, show bioactive properties. Ceramide and sphingosine-1-phosphate play a crucial role in the regulation of physio-pathological conditions. Small changes in their levels, in the ratio sphingosine-1-phosphate/ceramide as well as in chain length profiles of sphingolipids contribute to alter signaling pathways in neurons and glia, contributing to various neurological disorders. GM1 is considered a neurotrophic and neuroprotective compound and seems to be necessary for the correct functioning of neuronal membrane receptors, suggesting that a reduction in its level in the brain can be involved in neurodegenerative diseases. In this review, we give an overview of sphingolipid metabolism, summarizing the role of ceramide, sphingosine-1-phosphate, and GM1 in maintaining human health.

## 1. Sphingolipid Structure

**Figure 1:**
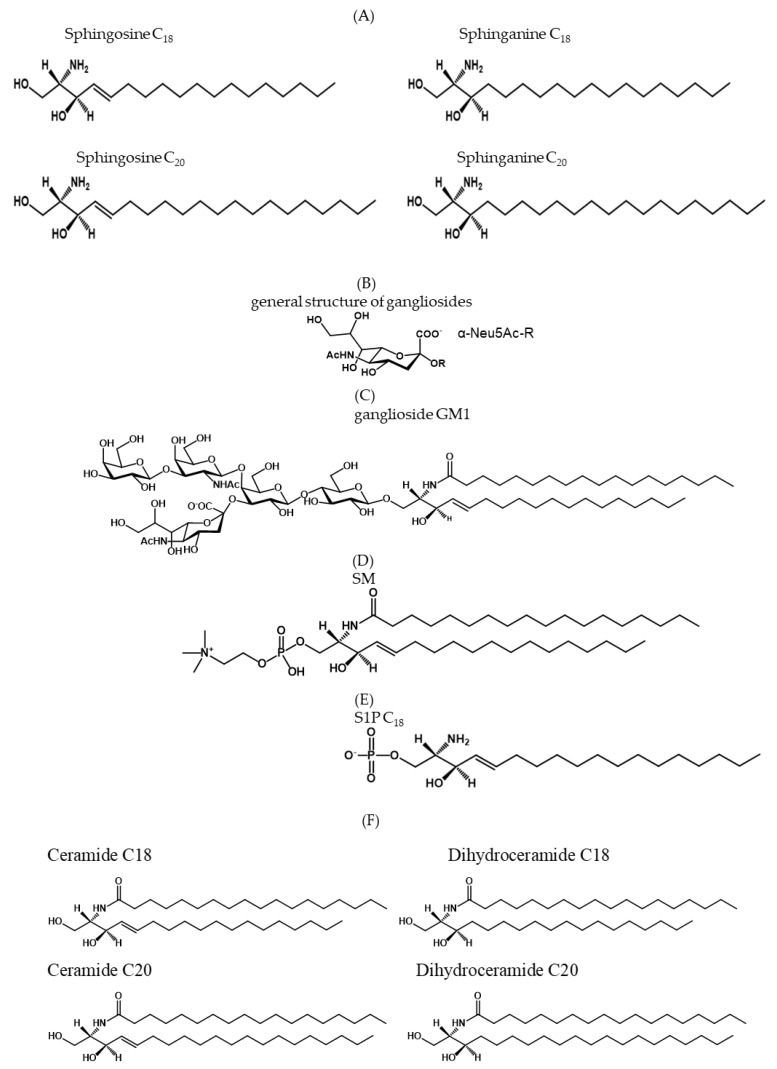
Chemical structure of sphingolipids. (**A**) structure of four long-chain bases present in sphingolipids. C_18_ and C_20_ sphingosines are the main long chain bases, covering 95% of total long chain bases. The C_18_ sphingosine and sphinganine are components of all sphingolipids, while the C_20_ species are present also in gangliosides; (**B**) general structure of gangliosides. The structure of sialic acid is represented linked to a general glycolipid R; (**C**) structure of ganglioside GM1; (**D**) structure of sphingomyelin; (**E**) Structure of C_18_ S1P; (**F**) chemical structures of C_18_ ceramide, C_20_ ceramide, C_18_ dihydroceramide and C_20_ dihydroceramide.

Sphingolipids are a complex and very large group of compounds present in all cells and tissues, some of which occur in high concentrations in the nervous system [1]. Sphingolipids are characterized by containing the long-chain amino alcohol 2-amino-1,3-dihydroxy-octadec (or eicos)-4-ene, commonly known as sphingosine (Figure 1A). The sphingolipid structure is very variable and originates from “de novo synthesis”, from the degradation of more complex structures [2], or the recycling of long chain bases via a salvage pathway [3,4].

Sphingolipids are crucial compounds in the physiology of cells, playing a role in membrane organization, membrane receptor activity and in modulating a wide range of physiological processes such as cell proliferation, differentiation and death.

Within the sphingolipids, there are the amphiphilic glycosphingolipids and phoshosphingolipids, in which the amino group of sphingosine is linked with a long acyl chain. Glycosphingolipids can be classified as neutral or acidic; the acidic ones contain sialic acid and are named gangliosides (Figure 1B,C), or contain sulphate and are named sulphatides. The main phosphosphingolipid is sphingomyelin (Figure 1D). In addition, as phosphosphingolipid we have in small amount sphingosine-1-phosphate (S1P) [3,5,6,7,8,9] (Figure 1E). Finally, we mention the very hydrophobic ceramide (Cer), a sphingosine which amino group is linked with a fatty acid (Figure 1F).

## 2. Sphingolipid Metabolism

The metabolic process leading to the final content and pattern of sphingolipids depends on several processes, namely biosynthesis, intracellular trafficking and catabolism. Being sphingolipids, membrane components, their structure and content are strictly associated with the turnover of the plasma membrane and depending on a two-way flow of molecules from and to the plasma membranes. This process is mainly driven by vesicular traffic, while non-vesicular transport via sphingolipid-binding proteins contributes significantly to specific steps [10,11,12,13,14,15,16].

De novo biosynthesis of sphingolipids initiates at the cytosolic leaflet of the endoplasmic reticulum (ER). The enzymes responsible for the reaction sequence that leads to the sequential reactions which produce ceramide, the common precursor of sphingomyelin and all glycosphingolipids, are also located in the ER. “De novo” synthesis of sphingolipids is catalyzed by serine palmitoyl transferase and by serine oleyl transferase [17], which condenses palmitoyl-CoA or stearolyl-CoA with L-serine to form dihydrosphingosine, named sphinganine [3,18]. Then, the N-acylation of sphinganine with a fatty acid produces dihydroceramide in a reaction catalysed by a family of six (dihydro)-ceramide synthases (CerS) [17,19]. In the salvage pathway, the same enzyme catalyses ceramide synthesis from sphingosine (Sph) [3], which is produced in the lysosomal catabolism of complex sphingolipids. In the “de novo” synthesis, Cer is formed by the addition of a double bond to dihydroceramide by dihydroceramide desaturase (DES). Ceramide reaches the Golgi apparatus by both the vesicular and ceramide transported CERT mediated pathways [13,14,15,16] for sphingomyelin (SM) synthesis, and by vesicular transport for the synthesis of glycosphingolipids. Cer, in turn, can be phosphorylated by Cer kinase to form Cer 1-phosphate (Cer1P) possibly via the direct action of CERT [3,20] or may be converted into complex sphingolipids (SM and glycosphingolipids). Cer1P is mainly synthesized in the Golgi apparatus. Cer1P is also present in the perinuclear region, implying that Cer kinase might also be localized close to the nuclear envelope, or that Cer1P could be transported from the Golgi apparatus to the nuclear membrane [21]. A specific Cer1P transfer protein (CPTP) has also been reported [22]. CPTP transfers Cer1P from the Golgi apparatus to the plasma membrane and to other organelles where it could exert its effects regulating different cell functions [22,23,24]. SM synthase (SMS existing in two isoforms) catalyse the synthesis of SM linking the 1-hydroxyl group of Cer to phosphocholine [3].

Regarding the synthesis of glycosphingolipids, ceramide is first glucosylated. A ceramide glucosyltransferase on the cytosolic surface of the early Golgi membrane generates glucosylceramide, the first glycosylated product, which can in minor quantities directly reach the plasma membrane [25], presumably transported through a non-vesicular mechanism. FAPP2 is a transfer protein for glucosylceramide that has a crucial role in the synthesis of complex glycosphingolipids [21,26,27,28,29]. A portion of ceramide is also transformed into galactosylceramide. Glucosylceramide is translocated to the luminal side of the Golgi, where the glycosyltransferases that synthesize more complex glycosphingolipids are located.

Both glycosphingolipids and sphingomyelin are transported from the Golgi apparatus to the plasma membrane through exocytotic vesicle-mediated traffic. According to this, the sphingolipid components of the inner membrane layer of vesicles, after fusion of these with the membrane, become components of the external plasma membrane.

**Figure 2:**
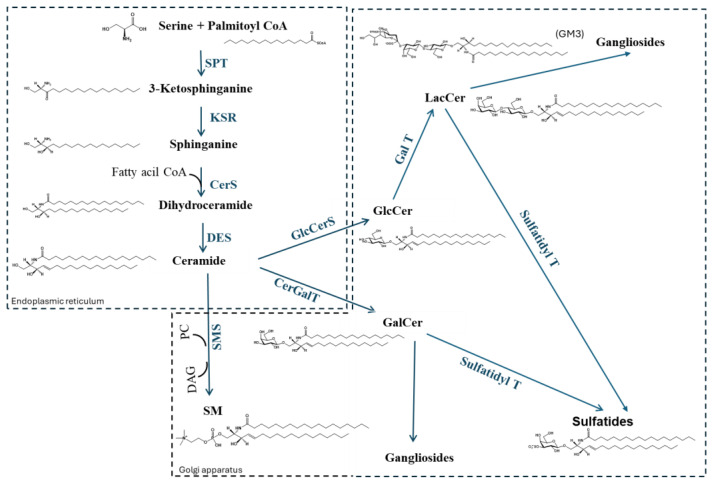
Sphingolipid synthesis and metabolism. SPT, serine-palmitoyl transferase; KSR, ketosphinganine reductase; CerS, ceramide synthase; DES, dihydroceramide desaturase; GlcCerS, glucosylceramide synthase; GlcCerDase, glucosylceramidase; CerGalT, ceramide galactosyl transferase; GalCerDase, galactosylceramidase; SMS, sphingomyelin synthase; Gal T, galactosyl transferase; Sulfatidyl T, Sulfatidyl transferase; SM, Sphingomyelin; GlcCer, Glucosylceramide; GalCer, Galactosylceramide; LacCer, Lactosylceramide; PC, phosphatidylcholine; DAG, diacylglycerol.

Very little is known about the regulation of the neo-biosynthetic process of sphingolipids. Glycosphingolipid production is believed to be mainly regulated transcriptionally by regulating glycosyltransferase or transporter protein levels. These have been in part confirmed by observing some parallel changes of cellular glycosphingolipid patterns and variations in the expression of the corresponding glycosyltransferases. However, it has been proposed that differential intracellular trafficking of different glycosphingolipids might affect the resulting glycosphingolipid patterns [30]. The general scheme for the synthesis of sphingolipids is reported in Figure 2.

**Figure 3:**
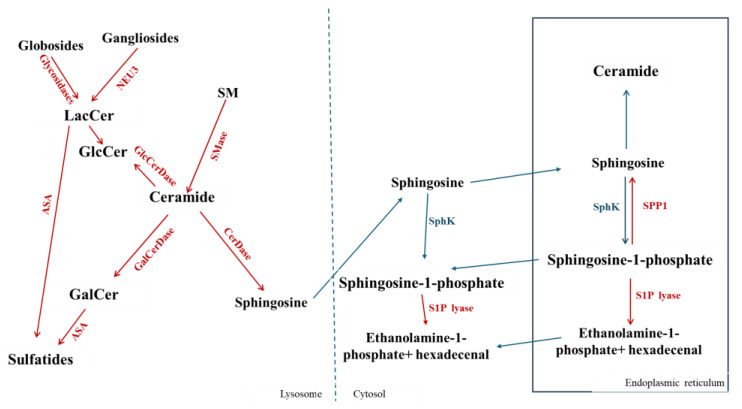
Sphingolipid catabolism. SM, Sphingomyelin; GlcCer, Glucosylceramide; GalCer, Galactosylceramide; LacCer, Lactosylceramide; SMase, sphingomyelinase; GlcCerDase, glucosylceramidase; CerDase, ceramidase; GalCerDase, galactosylceramidase; SphK, sphinosine kinase; SPP1, Sphingosine-1-Phosphate phosphatase; S1P lyase, Sphingosine-1-Phosphate lyase.

The degradation of plasma membrane sphingolipids takes place in the lysosomes, which are reached by the endocytic vesicular flow through the early and late endosomal compartments. Sphingolipids that start at the plasma membrane can travel to the Golgi on their way to lysosomes, be structurally modified there, and then be sent back to the plasma membrane. In neuronal cells observed in neuronal cells [31], this provides a further mechanism for controlling plasma membrane sphingolipid composition. Similarly, intermediate products of the lysosomal catabolism may exit the lysosomes and be recycled, through the salvage pathway, along the biosynthetic pathway [32]. In lysosomes, sphingolipids are sequentially degraded by specific hydrolases, in some cases assisted by specific activator proteins, to low-molecular-mass molecules, choline, sphingosine and the carbohydrate units. The carbohydrate units are recycled through several cellular processes. A scheme for the lysosomal degradation of sphingolipids is reported in Figure 3.

Cer can be de-acylated to Sph and fatty acids by three isoforms of ceramidases (classified according to their optimum pH) present at the level of membranes, lysosomes and mitochondria [3,33]. Sph, formed in the lysosomes, in the cytoplasm and in the ER, can be phosphorylated in an ATP-dependent reaction by sphingosine kinase to form S1P [34,35]. Two isoforms of sphingosine kinase have been demonstrated: sphingosine kinase 1 (SphK1) localized mainly in the cytosol and at the plasma membrane [36], and sphingosine kinase 2 (SphK2) found mainly at the plasma membrane and nucleus [37,38]. In the ER, S1P, in turn, may be phosphorylated or dephosphorylated to Sph by specific S1P phosphatases (SPPs) [39,40,41,42,43] or irreversibly cleaved by S1P lyase to form hexadecenal and phosphoethanolamine [44,45,46].

The biological role of simple degradation products derived from the lipid fraction of sphingolipids has been studied in detail, and the knowledge that some reactions involving ceramide occur in the plasma membrane has opened a new frontier in the regulation of sphingolipid membrane composition. Sphingolipid membrane composition depends not only on Golgi biosynthesis, lysosomal catabolism, and intracellular trafficking but also on direct transformations at the plasma membrane surface.

**Figure 4:**
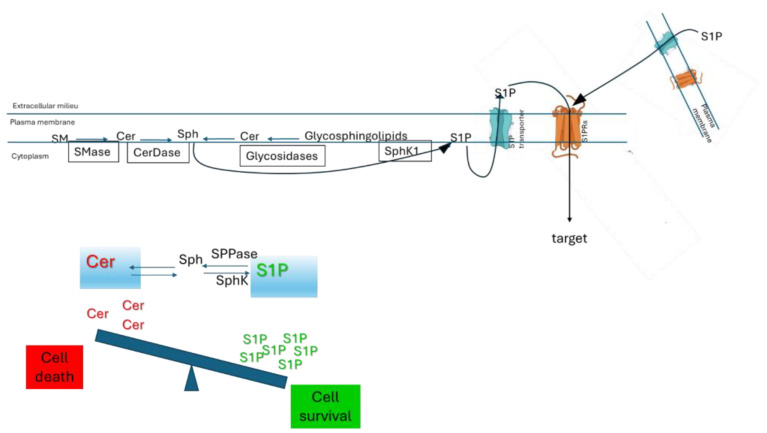
Sphingolipid rheostat. Schematic cartoon of important enzymes that regulate the S1P levels with “inside-out” signaling by the S1P/S1PR axis that can influence actions of the sphingolipid rheostat. SPPase, S1P phosphatase; S1PRs, S1P receptors.

Sphingomyelin hydrolysis by a plasma membrane Mg^2+^-dependent neutral sphingomyelinase (SMase) occurs at the cell surface [47]. The enzyme is membrane-resident or translocated to it from intracellular sites upon stimulus. A second enzyme [48,49] must also be considered: the lysosomal acid SMase, probably associated with the membrane after plasma membrane–lysosome fusion. A sphingomyelin synthase (SMS2) distinct from the Golgi isoform has been detected at the plasma membrane [50]. Therefore, the presence of two enzyme activities allows cells to adjust plasma membrane ceramide and sphingomyelin levels reciprocally in response to physiological changes, without having to sort substrates to intracellular metabolic compartments (Figure 4).

Comparable observations have been documented for additional enzymes involved in bioactive sphingoid regulation; plasma membrane-associated ceramidases and SphK1 have been implicated in producing sphingosine and/or sphingosine-1-phosphate at the cell surface [51,52,53]. S1P can act intracellularly or can be transported across the membrane by specific transporters and can act through specific receptors (S1PRs) present at the plasma membrane [54]. Sphingosine can also be phosphorylated to form S1P by sphingosine kinase 2 (SphK2) at the mitochondria, nucleus, and ER [55] (Figure 4).

With respect to glycosphingolipids, the information on the plasma membrane regulation of ganglioside content by desialylation–sialylation reactions goes back many years. The existence of a membrane-associated sialidase has been previously known [56,57]. However, more recent reports confirm the existence of sialidase Neu3, which differs from the lysosomal sialidase [58,59]. More recent is the information of the plasma association of acidic glucosidase, galactosidase and hexosaminidase, due to a lysosome–plasma membrane fusion process. In addition, the plasma membrane gluocerebrosidase GBA2, different from the lysosomal GBA1, has been studied in detail. These glycosidases, together with the plasma membrane sialyltransferase as well as other glycosyltransferases, are [60] associated with the plasma membrane, allowing a series of equilibrium reactions involving the glycosphingolipid structures and ceramide [61] (Figure 4).

## 3. The Hydrophobic Sphingolipids

### 3.1. Ceramide and Ceramide Properties

Ceramide is characterized by a long-chain sphingoid base *N*-acylated with fatty acids of varying chain lengths (usually C14–C26) [62]. In mammals, ceramide synthases are the enzymes of *N*-acylation of sphinganine/sphingosine and each of the six ceramide synthases (CerS1–6) mainly use as substrate a small range of acyl-CoAs, giving rise to different ceramide species [19]. The six endoplasmic reticulum (ER) membrane-bound CerS each N-acylate substrates using a select group of fatty acyl-CoAs, but they exhibit less specificity in the utilization of long-chain bases. It is not yet known why mammals have multiple CerS that generate a specific subset of ceramide, but this suggests a pivotal role for ceramide with specific fatty acid moieties in cell physiology [63]. Since sphingolipids are particularly abundant in nervous tissue, small changes in chain length profiles can profoundly alter membrane properties and signaling pathways in neurons and glia [64].

It has been demonstrated that the different molecular species of Cer have a different effect on the membrane organization; the acyl chain saturation drives the membrane lateral organization, and the chain asymmetry dictates interdigitation and membrane morphology [64]. The saturated ceramide have a crucial role in increasing the order and promoting gel/fluid phase separation in the membranes. On the contrary, unsaturated ceramides show a reduced ability (C24:1) or no ability (C18:1) to form gel domains at 37 °C [64]. On the other hand, the variation among saturated species is reduced, and these differences are generally associated with the morphology and size of the gel domains. Moreover, very long chain ceramides form tubular structures [64].

In the CNS, the different isoforms of CerS are characterized by specific expressions. In particular CerS1, that utilizes mainly C18-Cer, is highly expressed in neurons of the forebrain and cerebellum; CerS2, that utilizes mainly C22-24-Cer, is enriched in oligodendrocytes and myelin, where very-long-chain ceramides stabilize compact myelin membranes; CerS5/6, that utilizes mainly (C16-Cer), is widely expressed, including in astrocytes and microglia, and is often linked to stress responses; and CerS3, that utilizes mainly C24–26-Cer, is expressed at low levels in brain except in reproductive tissues, but contributes to minor pools in glia [63,65].

Different studies have indicated that some functions of ceramides are chain length dependent, and chain length guides membrane and signaling functions C16 Cer is a potent inducer of apoptosis and inflammatory cytokines; C18 Cer promotes synaptic plasticity and neurite outgrowth by organizing lipid rafts and recruiting signaling kinases at growth cones; very-long-chain ceramides (C22–24) enhance membrane order and rigidity, supporting myelin integrity and long-term axonal conduction [63,65].

Cer is a bioactive lipid able to act as second messengers [33,66,67,68,69] activating protein phosphatase 2A (PP2A), atypical PKCζ, and stress-activated MAP kinases, but inhibiting Akt [70,71,72,73]. Cer is involved in the regulation of cell fate participating in the regulation of numerous cellular functions, including the modulation of stress resistance, proliferation, differentiation of nervous system cells to mature phenotypes [74,75,76], cell death, senescence, adhesion, migration, inflammatory responses, angiogenesis, and intracellular trafficking within the central nervous system [77,78]. In lower organism models such as *Caenorhabditis elegans* and yeast, a crucial role of sphingolipids in aging has been shown, demonstrating a link between Cer synthesis and longevity [6,75,79,80,81,82]. Cer was demonstrated to be a negative modulator of proliferation and survival in neurons and astrocytes and to be involved in regulating neuronal differentiation [83,84,85]. Human iPSC-derived neurons and glia differ in ceramide synthesis, in the composition of ceramide isoforms, and in the responses to altered ceramide levels. In particular, studies demonstrated an increase of the de novo synthesis of Cer in glia, especially very-long-chain species, and that glia tolerates high ceramide loads; on the contrary, neurons synthesize less Cer but are much more sensitive to small ceramide increases, leading to ER stress and apoptosis [86]. In Alzheimer’s disease (AD) patients, there is an increase in Cer levels. These variations are associated with inflammation and neuronal death [87]. Aβ increases Cer, up-regulating the activity of sphingomyelinases (SMases) [88,89,90] or of serine palmitoyltranferase (SPT) activity [88,91]. Analysis of human post-mortem brain material demonstrated that Alzheimer’s disease (AD) patients show an increase in C16-Cer and altered C24-Cer/C18-Cer ratios in membranes, contributing to synaptic loss and neuro-inflammation [91]. In the early stages of AD, levels of Cer C22:0 and C24:0 derived from de novo synthesis increased [92]. Moreover, it has been shown that in AD there is an up-regulation of *d*18:1 species of sphingolipids, but recently it has been shown that AD is associated with distinct changes of plasma Cer, such as upregulation of Cer *d*18:1/16:0 [93]. Furthermore, in the brain, in particular in the myelin sheath, SM is a very abundant sphingolipid [2]; the SM C16:1, SM C18:1 and SM C16:0 and one hydroxy-sphingomyelin SM (OH) C14:1 were found at very high levels in AD brains [87,94]. Fumonisin B1, a ceramide synthase inhibitor, affects neurons in vitro, causing equine leukoencephalomalacia, and a failure of neural tube closure [35,95,96,97,98,99]. These changes in ceramide metabolism are believed to play a role in the disease’s progression and severity. Furthermore, higher Cer levels have been observed in Parkinson’s disease (PD) patients with cognitive impairment compared to controls without cognitive impairment, and there is and direct correlation between high Cer levels and worse cognitive function [100]. The total amount of ceramide in the anterior cingulate cortex of brains from Parkinson’s disease (PD) cases was significantly lower than in the anterior cingulate cortex of control cases. Most individual ceramide species were decreased in the anterior cingulate cortex samples of PD cases, but the highest reductions were found in Cer(d18:1/22:0), Cer(d18:1/23:0), and Cer(d18:1/24:1) [101]. Alterations in Cer metabolism are fundamental elements in several tumors, including glioblastoma. An inverse correlation between Cer levels and malignant progression and poor prognosis of glioblastoma has been reported [102]. Moreover, in U87MG glioma cells, the levels of the epidermal growth factor receptor variant III (EGFRvIII), generally expressed by glioblastoma, confer a resistance phenotype to the alkylating agent Temozolomide by counteracting Cer increase [103]. Moreover, in glioma cells overexpressing EGFR resistant to TMZ, the treatment with the drug increased Cer levels, including very-long-chain Cer C22:0 and C24:0, to a lesser extent compared to the sensitive cells [103].

Ischemic injury also increases Cer in vulnerable neurons. Elevated ceramide levels have been observed in association with reperfusion after an ischemic event [104]. After mild ischemia–reperfusion, it has been shown that ceramide species accumulate in isolated mitochondria and the ER [105]. Mitochondrial Cer elevation was limited to C18:0-, C18:1 and C16:0 species, whereas the ER displayed broad ceramide accumulation, consistent with compartment-specific activation of ceramide synthases [106]. In mice, the decrease of C22-Cer in myelin, due to the lack of CerS2 activity, causes damage in sheath stability [107].

A dihydroceramide desaturase 1 (DES1), responsible for the conversion of dihydroceramide (dhCer) to Cer, low enzymatic activity leads to accumulation of dhCer and other dihydrosphingolipids. Changes in the ratio between dhCer and Cer has a crucial effect in vitro and in vivo [108]. A correlation between high levels of dhCer and neurodegenerative diseases such as PD and AD has been proposed; in patients with clinical signs of AD the levels of dhCer in cerebrospinal fluid were significantly higher than in people with mild cognitive impairment and dhCer levels in plasma or serum is negatively correlated with the prevalence and incidence of AD [109,110].

The role of DES1 and dhCer in the nervous system is further highlighted by data in the literature demonstrating that loss-of-function variants in *DES1* are associated with hypomyelinating leukodystrophy and neurodegeneration [111,112,113,114]. Moreover, the inhibition of dhydroceramide desaturase 1 promotes autophagy, influencing the final cell fate in glioblastoma cells [115].

### 3.2. Sphingosine-1-Phosphate and Its Properties

S1P is involved in the regulation of a variety of biological functions; its levels have to be tightly regulated through the control of the different enzymes responsible for S1P synthesis and/or degradation [33,77,116]. S1P regulates crucial aspects of cell fate, including apoptosis suppression [117], cell growth regulation [118], calcium homeostasis [119], and cell trafficking and cell signaling. S1P acts mainly through the binding to a family of 5 G-protein coupled S1P receptors (S1PR1-5). In the nervous system, S1PR1, S1PR2, S1PR3 and S1PR5 are expressed, supporting the crucial role of S1P signaling [120,121,122,123,124,125]. S1P is a bioactive molecule acting as a signaling molecule able to regulate different physiological and pathophysiological conditions [69,121,126,127]. In particular, S1P activates complex cascades responsible for the regulation of different cellular processes such as cell migration and angiogenesis [122,128]. S1P increases vascular density, amplifies VEGF-induced angiogenesis and activates Rac/Rho signaling, stimulating the assembly of adherens junctions on endothelial cells [128,129,130].

Indeed, S1P has fundamental roles in the CNS, including neurogenesis, neuroprotection, neuronal repair, and remyelination, among others [35,131]. S1P, in both neurons and glial cells, is a positive modulator of cell proliferation, survival, and motility [132]. Moreover, S1P induces calcium signaling in cerebellar astrocytes, while differentiated neurons lack a calcium response to S1P. This mechanism could participate in neuron–glia interactions within the cerebellum [133].

The S1P plays a crucial regulatory role in the nervous system, acting with the opposite role of Cer with the known concept of the “Cer/S1P rheostat” (Figure 4). The variation in S1P levels leads to pathological conditions, contributing to various neurological disorders. S1P has different functions in the nervous system; in particular it is involved in: (i) neuroprotection and cell survival; S1P, acting through its G-protein-coupled receptors (S1PR1–5), activates anti-apoptotic signaling pathways promoting cell proliferation, migration, and cytoskeletal dynamics, giving the chance to neurons and glial cells to overcome injury or stress [134,135,136]; (ii) myelination and remyelination; S1P is involved in protecting oligondendrocytes responsible of myelin formation [137,138]; the inhibitor of S1PRs, fingolimod, is already a drug in use on multiple sclerosis (MS) [55,139,140,141]; (iii) neuroinflammation modulation; S1P in pathologies such as AD and PD, S1P, depending on the subtype of activated S1P receptor, may suppress or promote inflammatory responses [142]; (iiii) neurodegenerative diseases; alterations in S1P metabolism may promote the disease progression affecting autophagy, mitochondrial function, and neuronal resilience [143,144,145,146].

Aβ production has a direct correlation with reduced expression of sphingosine-kinase 1 and enhanced expression of S1P lyase. In AD, fingolimod decreases neuronal a generation [147] and the injection of fingolimod in the hippocampus of a rat model with pre-aggregated Aβ reduces hippocampal neuron damage [148]. In PD the gene expression of sphingosine kinases and of S1P lyase was significantly modified and the enzymatic activity of SphK1 and SphK2 were significantly decreased [149,150,151,152]. In particular, in PD in vitro models, SphK inhibition was associated with elevated α-synuclein secretion, decreased PI3K/Akt pathway activation, and upregulation of pro-apoptotic genes [150]. S1P is known to be an onco-promoter molecule in many tumors, as well as glioblastoma [34,153,154,155]. Several studies demonstrate that, in glioblastoma cells, S1P induces proliferation, motility and invasiveness promoting the malignant behavior [156,157,158]. Increased level of S1P produced by SphK1 is related to the oncogenic effects regulated by S1P and this is correlated to high level of SphK1 in glioblastoma cell lines [159,160]; the inhibition of SphK1 have been shown to decrease xenografts and human glioblastoma cell growth [161]. The expression of SphK1 in glioblastoma is inversely correlated with patient survival [162]. Glioblastoma cells overexpressing EGFR-VIII are characterized by higher levels of extracellular S1P and increased sphingosine kinase-1 (SK1) activity than empty vector expressing cells and the EGFR overexpressing cells are resistant to TMZ and have higher invasiveness properties compared to the empty vector expressing cell [66,163].

## 4. The Amphiphilic Gangliosides

Gangliosides, containing up to 5–6 sialic acid units, are enriched tenfold in neurons relative to non-neuronal cells. 3–4% of the total cellular gangliosides are present in the cytosol where they interact with soluble proteins [164]. However, the majority of them are present on the outer layer of plasma membranes. Inside the membrane, gangliosides exert strong interactions with the neighboring membrane hydrophobic components, participating in the membrane stabilization and organization. The ganglioside large hydrophilic oligosaccharide chain, together with the net of hydrogen bonds formed by their ceramide moiety, determines chemical and physico-chemical properties in favor of the formation of membrane lipid domains, called lipid rafts [165], that are rigid portions of the membranes. During human brain aging, the 3-keto-sphinganine synthase shows an increasing specificity for stearoyl-CoA compared with palmitoyl-CoA; consequently, the ratio of sphingolipid C20 and C18 sphingosines increases [166]. As a result, the gangliosides become more hydrophobic and, consequently, the plasma membranes and the lipid rafts become more rigid during aging. However, currently no information is available on the biological role of these changes.

The ganglioside oligosaccharide protrudes from the outer layer of the membrane and can interact with the hydrophilic portion of the membrane’s neighboring proteins. The interactions between gangliosides and membrane proteins, such as membrane receptors, take place at the lipid–water interface and are driven by saccharide-saccharide or by sialic acid-positively charged amino acid interactions.

Cellular ganglioside content is determined by a complex network of metabolic pathways, including biosynthesis in the Golgi apparatus, catabolism in lysosomes and intracellular trafficking [167]. In addition to this, gangliosides can be structurally rearranged at the cell surface, by membrane-associated glycosidases [168], among which the sialidase Neu3 [169] plays a key role in regulating the amount of sialidase resistant monosialogangliosides and lactoisytlceramide produced from polysialylated gangliosides.

During neuronal differentiation, ganglioside levels rise progressively, shifting from simple forms to complex and polysialylated gangliosides. Ganglioside composition and levels do not change over a long period, but some brain regions in elderly people show reduced GM1 and GD1a [170]. The reduction of the GM1 ganglioside has been associated with a number of neurological diseases.

### Ganglioside GM1 and Its Properties

GM1 (Figure 1C) is one of the major gangliosides in the mammalian brain. It is a monosialoganglioside that, according to the IUPAC-IUB nomenclature, is correctly coded as Neu5AcII^3^GgOse_4_Cer. GM1 covers 10–15% of total brain ganglioside content and is largely associated with membrane lipid rafts. The ceramide moiety of GM1 contains over 95% of C18 sphingosine at birth, the remaining being the sphinganine species. With aging, the ganglioside is progressively synthesized with ceramide that contains the C20 species. In the brains of old mammals, the C20 species are predominant against the C18 species [171].

**Figure 5:**
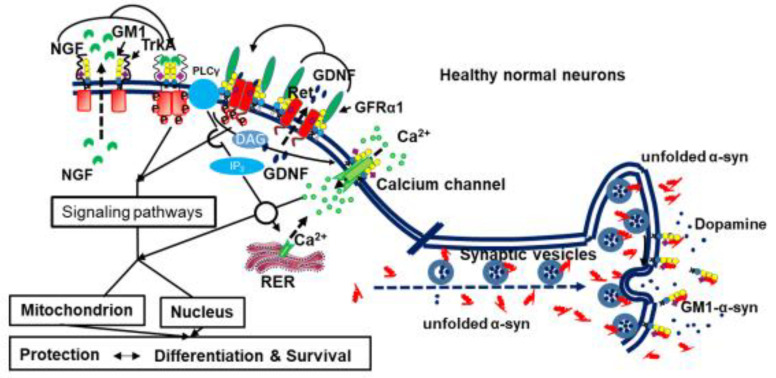
GM1 as a neuroprotective and neurotrophic factor. Schematic cartoon of the role of GM1 in activating neuronal receptor functions.

GM1 has been deeply studied for its biological properties, and it is now considered a neurotrophic and neuroprotective compound [172] (Figure 5).

The neurotrophic nerve growth factor, NGF, is necessary for the activity of the trans-membrane tropomyosin receptor kinase A, TrkA, encoded in humans by the *NTRK1* gene.

NGF binding to TrkA induces receptor dimerization, associated with conformational changes of the receptor cytosolic part and to the auto-catalytic kinase activity of this portion. Phosphorylated tyrosine residues in TrkA’s cytoplasmic domain recruit signaling molecules, initiating a cytosolic phosphorylation cascade that activates signaling pathways, such as the Ras/MAPK and the PI3K/Akt, which are required for neuronal differentiation, maintenance and survival.

Nevertheless, the NGF-TrkA system requires the GM1 ganglioside and the formation of the NGF-TrkA-GM1 tri-component complex to turn on the neuronal signaling. PC12 cells contain very little GM1 in their plasma membranes and the exogenous administration of GM1 markedly potentiates NGF-mediated TrkA activation; in cells lacking endogenous GM1, NGF failed to induce TrkA auto-phosphorylation of Trka, but restoring GM1 levels reinstated TrkA responsiveness to its ligand [173]; GM1 does not substitute NGF, which continues to be required for receptor functions. Thus, GM1 is required for the proper functioning of TrkA and supports the knowledge on the neurotrophic and protective effects mediated by the ganglioside GM1.

If the autophosphorylation of TrkA requires the tricomponent complex NGF-TrkA-GM1, TrkA and GM1 should co-localize. GM1 is a component of the lipid rafts in cultured cells and in brain cells [174,175,176,177]. Conversely, in Neuro2a (N2a) neuroblastoma cells, although TrkA activation and their differentiation require both NGF and GM1, TrkA is located in a fluid portion of the plasma membrane separate from the GM1 lipid raft. The GM1 oligosaccharide binds to the extracellular domain of TrkA, while the GM1 ceramide is located well away from the receptor membrane domain. It has been suggested that the extracellular portion of the TrkA located outside the GM1 lipid raft may flop down onto the plasma membrane, approaching the GM1 oligosaccharide chain [178]. A GM1-binding site in the extracellular domain of TrkA implies that the GM1 oligosaccharide could act as an endogenous activator of the TrkA receptor. The capability of the GM1 oligosaccharide alone to induce neuritogenesis in a neuroblastoma cell line was observed in the past [179] and has been recently confirmed [180]. It has been suggested that the specific carbohydrate pattern of GM1-oligosaccharide links NGF and the TrkA receptor, directly promoting and stabilizing their interaction and thereby inducing TrkA phosphorylation and MAPK signaling [178,181,182].

The neurotrophine GDNF is necessary for the activity of RET (rearranged during transfection). RET is a tyrosine kinase receptor, which interacts with the glial cell line-derived neurotrophic factor GDNF receptor GFRα1, a GPI-anchored membrane protein. This interaction promotes proliferation, differentiation, survival, migration, and metabolism via the PI3-K/Akt and Src signaling pathways. RET is required for the development of both the peripheral and central nervous systems and is regarded as necessary for the survival of adult dopaminergic neurons.

GDNF, a soluble neurotrophine, binds GFRα1 with high affinity, inducing RET to redistribute into lipid rafts [183] and undergo auto-phosphorylation. GFRα1-GDNF-RET trimeric complex is the trigger for neuronal signaling linked to mitochondrial activity and cell survival. The transcription factor Nurr1 controls RET expression at the membrane, and Nurr1 levels are decreased when α-synuclein is overexpressed [184].

GM1 is required for RET auto-phosphorylation, and the subsequent phosphorylation cascade is essential for downstream signaling. Moreover, GM1 prevents the formation of α-synuclein aggregates [185,186]; these aggregates suppress Nurr1, a potent activator of RET.

GM1 is necessary for the activation of RET. A mouse model was obtained by disruption of the *B4galnt1* gene. The omozygote mice had no GM1, and the heterozygotes had a minor quantity. The mice showed severe and mild neurodegenerative phenotypes, respectively, characterized by abundant α-synuclein and aggregates alongside decreased RET and phosphorylated RET expression [187]. Injection of a membrane-permeable analog of GM1, capable of partially crossing the blood–brain barrier [188,189,190,191], to these mice partially restored the correct neuronal functions. When added to striatal slices in situ, GM1increased phosphorylated RET levels and downstream cell signaling in a concentration- and time-dependent manner [192].

Although the precise role of GM1 in regulating GFRα1-GDNF-RET signaling remains unclear, GM1 is clearly necessary for RET activation and is suggested to play a specific role in avoiding α-synuclein aggregation.

α-Synuclein, a cytosolic disordered protein, is abundant in neurons and concentrated in presynaptic endings where it displays high affinity for negatively charged membrane lipids [193] thereby thus promoting synaptic vesicle docking to the membrane. This suggests its role in the fusion of synaptic vesicles with membranes [194].

Under certain conditions, the soluble disordered protein assembles into oligomers that quickly grow in molecular mass, producing large insoluble fibrils found in intracellular and intercellular Lewy bodies [195].

GM1 strongly binds to α-synuclein [185,186,195], maintaining its non-amyloid α-helix structure and thus providing neuroprotection. Treating the *B4galnt1* heterozygote mice with the GM1 oligosaccharide produced similar results [196]. When GM1 was injected into rats overexpressing human mutant α-synuclein (A53T), striatal α-synuclein aggregation was reduced and the nigrostriatal system showed neurorestorative improvements [197,198].

Axon terminal membranes contain high concentrations of both GM1 and α-synuclein but α-synuclein is concentrated at the cytoplasmic side of the plasma membrane [199], while GM1 is inserted into the outer layer of the membrane. Thus, the interaction between GM1 and α-synuclein must occur after the exit of α-synuclein from the axon terminal membrane. While this occurs, GM1’s interaction with α-synuclein inhibits protein aggregation [200]. Lipid domains are structurally rigid yet highly dynamic platforms, and any change in the component content requires a rearrangement of their composition [201]. Any process that reduces the Golgi neo-synthesis of GM1 or the plasma membrane conversion of GD1a into GM1, hereby making lipid rafts less rigid, could dramatically reduce the interaction between GM1 and α-synuclein. Then α-synuclein would start aggregating, and the aggregates would be transported to other cells, i.e., entering into the post-synapse and neural body, as well as into other brain cells.

However, a second cell location for the GM1-α-synuclein interaction should be taken into account. A minor fraction of GM1 complexed with proteins is found in the cytoplasm. We could therefore speculate that the small amount of cytosolic GM1 is responsible for stabilizing α-synuclein and inhibiting its aggregation. Only a small number of studies address cytoplasmic GM1 and its associated protein. Using photolabeling and crosslinking techniques, cytoplasmic proteins associated with GM1 were identified in human fibroblasts [202]. Specifically, a set of several specific protein bands with a molecular mass between 30 kDa and approximately 100 kDa was identified. Unfortunately, under physiological conditions, fibroblasts contain very low levels of α-synuclein, and no photolabeling studies were performed on the neuron cytoplasm.

The necessity of GM1 for the correct function of neuronal membrane receptors suggests that any reduction of brain GM1 can be involved in neurodegenerative diseases. The role of GM1 in the pathogenesis of PD characterized by fibrillary α-synuclein aggregation and progressive degeneration of nigro-striatal dopaminergic neurons has been studied. The main result of this is that in the PD cell brain cytosol, aggregates composed of multiple substances, known as Lewy bodies, are present. The Lewy bodies contain aggregates of α-synuclein. α-Synuclein, is toxic in its aggregate form. In addition, in this, the progressive death of dopaminergic neurons leads to the progressive reduction of dopamine.

Genetic alterations are linked to PD, some of which involve glycosphingolipids. Approximately 5% of PD cases result from reduced expression of the enzyme GBA1, the lysosomal glucocerebrosidase. This results in the accumulation of glucosylceramide followed by progressive neurodegeneration. It has long been recognized that patients with type-1 Gaucher’s disease, who have partially reduced GBA1 activity but no serious neurological symptoms, gradually develop PD. The rest of PD cases are sporadic and linked to various genetic errors associated with glycosyltransferases and observed in parents at the age of 60 or older. Aging in humans has been linked to a reduction in the ganglioside GM1 and more complex gangliosides. The decrease in ganglioside GM1 under a specific threshold level seems responsible for the aetiopathogenesis of the sporadic form of PD [203,204,205,206]. In particular, expression of *the B3galt4* and *St3gal2* genes is markedly reduced. The *B3galt4* gene encodes the galactosyltransferase that converts GM2 into GM1, while the *St3gal2* gene encodes the sialyltransferase that synthesizes GM1b from tetrahexosylceramide, GD1a from GM1a, and GT1b from GD1b. Since glycosyltransferases act sequentially, reduced expression of the *B3galt* and *St3gal2* results in a partial decrease ofGM1. The absence of *B4galnt1* gene expression in a PD mouse model, which controls the synthesis of GM1 from GM2, leads to loss of GM1 and elevated levels of GM3, GD3 and GM2 gangliosides. These mice show very serious neurodegeneration, and their life is very short. Nevertheless, the heterozygous loss of the *B4galnt1* gene leads to a partial decrease in GM1 similar to that observed in PD patients [187,188,204,205,206,207] and can be used for studies of replacement therapy using GM1. Injection of GM1 to the heterozygous mice reduced the non-neurological gastrointestinal and sympathetic cardiac symptoms that are characteristic of PD [208]. Less evident was the improvement in the neurological symptoms. This is unsurprising, since only a minimal fraction of GM1 is known to cross the blood–brain barrier [209], thereby limiting its ultimate benefits. Nevertheless, superior effects on the neurological symptoms were observed with a more hydrophobic synthetic GM1 analog, which penetrates the brain in greater amounts [188,189,190,191]. However, because of its significant toxicity, this compound cannot be used as a therapeutic agent.

To address these issues, animals were administered a soluble form of GM1 oligosaccharide. As we reported above, GM1 binds the neuronal receptor through its oligosaccharide chain, and all the neurotrophic and neuroprotective effects exerted by GM1 were replicated using its free and soluble oligosaccharide [210]. The GM1 oligosaccharide crosses the blood–brain barrier and reaches the brain neurons [209]. Administration of the GM1 oligosaccharide to heterozygous mice fully reversed physical symptoms, decreased α-synuclein aggregation and normalized tyrosine hydroxylase expression and neurotransmitter concentrations in the substantia nigra, restoring the wild-type healthy condition.

These findings are consistent with GM1’s reported neurorestorative and neuroprotective effects in additional in vivo PD models, including mice and non-human primates exposed to 1-methyl-4-phenyl-1,2,3,6-tetrahydropyridine [197] and rats transduced with human A53T mutant α-synuclein via adeno-associated viral vector [211].

## 5. Conclusions

Sphingolipids play a crucial role in membrane organization, membrane receptor activity and in modulating cell fate. Cer and S1P play a pivotal role in the regulation of physio-pathological conditions within the central nervous system [74,75,76,77,78]. Small changes in their levels, in the sphingosine-1-phosphate/ceramide ratio and in sphingolipid chain length profiles alter signaling pathways in neurons and glia and contribute to various neurological disorders. It is known from the literature that ceramide chain length drives different signaling functions [62,63,64,65]. In AD patients, there is an increase in Cer levels that is associated with inflammation and neuronal death [87] and in PD patients, it has been demonstrated a direct correlation between high Cer levels and worse cognitive function [100]. Furthermore, it is known to be a correlation between high levels of dhCer and neurodegenerative diseases such as PD and AD [109,110]. The variations in S1P level lead to pathological conditions, contributing to various neurological disorders such as myelination and remyelination [137,138,139,140,141], neuroinflammation modulation [142], and neurodegenerative diseases [143,144,145,146].

In several tumors, including glioblastomas, the alterations in Cer and S1P metabolism are a crucial element. It has been demonstrated an inverse correlation between Cer levels and malignant progression and poor prognosis of glioblastoma [102]. S1P is recognized to be an onco-promoter molecule in many tumors, as well as glioblastoma [34,153,154,155].

GM1, a neurotrophic and neuroprotective compound, has been suggested as a necessary factor for the correct function of neuronal membrane receptors [172]. This suggests that the reduction of its level in the brain can be involved in neurodegenerative diseases. GM1 stabilizes the non-amyloidogenic α-helix conformation of the protein, strongly interacting with α-synuclein [185,186,195], thus exerting a neuroprotective effect. The necessity of GM1 for the correct function of neuronal membrane receptors suggests that any reduction of brain GM1 can be involved in neurodegenerative diseases.

Altogether, this data suggests that the dysregulation of sphingolipid metabolism and levels is deeply involved in pathological conditions of the nervous system such as neurodegenerative disorders and cancer [211,212,213,214]. These data on the altered sphingolipid metabolism suggest that its regulation may help to identify targets for future therapeutic interventions.

## Data Availability

Not applicable.
